# Pyoderma gangrenosum after hip hemiarthroplasty; a case report

**DOI:** 10.1016/j.tcr.2022.100689

**Published:** 2022-08-01

**Authors:** E. Laskaratou, N. Trygonis, R. Dimitriou, G. Kouvidis

**Affiliations:** Department of Orthopaedics, University Hospital of Heraklion, 71500 Crete, Greece

**Keywords:** Pyoderma gangrenosum, Hip hemiarthroplasty, Mortality, Hip fracture

## Abstract

Pyoderma gangrenosum is a severe and rare neutrophilic disorder that can present as a complication following any kind of surgery, usually after breast and abdominal surgery. This condition mimics infection, delaying prompt diagnosis and appropriate treatment with high dose of corticosteroids. We describe a case of pyoderma gangrenosum after hip hemiarthroplasty, in an 86-year-old woman, who sustained a neck of femur fracture after a simple fall. The patient was diagnosed 2 weeks postoperatively with pyoderma gangrenosum through a biopsy with clinical manifestations from other systems such as seizures and atrial fibrillation.

## Introduction

Pyoderma gangrenosum (PG) is a rare neutrophilic dermatosis (ND) characterized by rapidly evolving painful ulcers, with undermined borders and peripheral oedema (red skin). Epidemiological studies indicate that the average age of PG onset is in the mid-40s, with an incidence of a few cases per million person-years. PG is often associated with a variety of other immune-mediated diseases, most commonly inflammatory bowel disease and rheumatoid arthritis [1]. The pathophysiology of PG remains incompletely understood. It represents a complex reaction pattern with either multiple pathways or the convergence of various features that create a heterogeneous disease with variant presentation and course, involving dysregulation of the innate immune system, as well as abnormal chemotaxis, neutrophil migration, phagocytosis, bactericidal ability, and abnormal neutrophil trafficking [2]. Cases of PG have also been reported as a response to a minor or major trauma. A recent literature review found 220 cases of postsurgical PG, all surgical specialties included, with 25 % in plastic and reconstructive surgery, 14 % in cardiothoracic surgery, 14 % in digestive surgery, 13 % in obstetrics and gynaecology, and 12 % in orthopaedics [3]. Although the mechanisms that are responsible for a pathergic phenomenon are unknown, a skin injury that is observed in this phenomenon can provoke an inflammatory response which is much more intense and extensive than in a normal skin, leading to an extensive release of cytokines from keratinocytes and other cells in the epidermis and dermis and in a perivascular infiltration.

Clinically, there are five distinct clinical variants that are recognized, which include classic, bullous, vegetative, pustular, and peristomal PG. Lower extremities are most commonly affected, while rare locations such as the genital and perianal area have been reported in infants [4]. PG is considered a diagnosis of exclusion, when there is an absence of specific histopathological and laboratory criteria. PG is presented as a rapidly growing, painful ulcer with a purulent cover, and that is why it can mimic an infection [5]. There is no standard medical treatment, although high dose of corticosteroids can impede the development of the ulcer.

We present a case of pyoderma gangrenosum occurring in an 86-year-old woman, who underwent a hip hemiarthroplasty, following a fracture of the left femoral neck.

## Case report

**Fig. 1:**
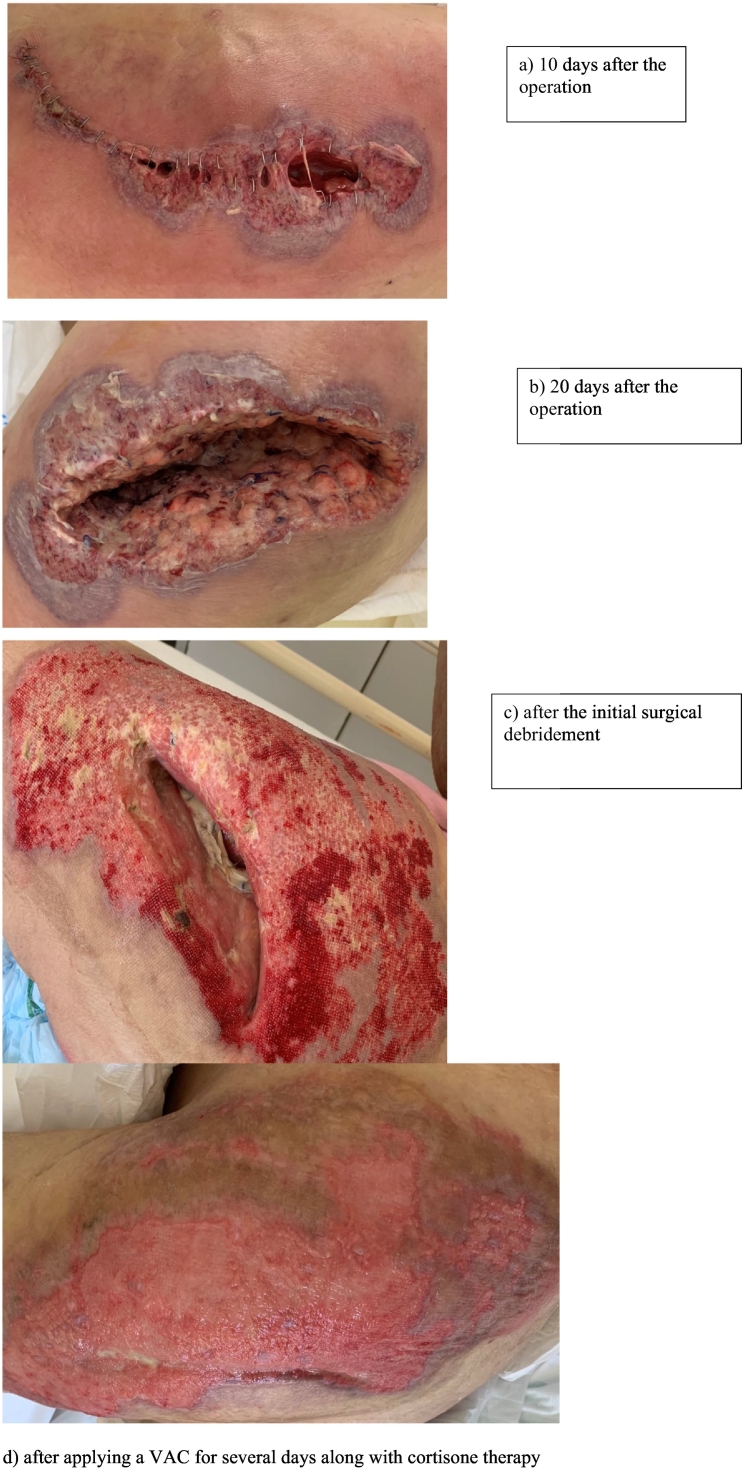
a) 10 days after the operation, b) 20 days after the operation, c) after the initial surgical debridement, d) after applying a VAC for several days along with cortisone therapy.

An 86-year-old woman underwent a cemented hip hemiarthroplasty, following a fracture of the femoral neck classified as Garden III. On the third post-operative day, the patient complained of swelling of her left leg. Her temperature was 37.8 °C. A triplex ultrasound of the left leg was performed and deep vein thrombosis was excluded. Gradually, a dehiscence of the surgical wound was noted. Her inflammatory markers were raised with white blood cell count of 14,300/ml (normal range 5000–10,000), platelets-PLT of 450,000/μl (normal range 150,000-450,000/μl) as well as a gradual elevation of CRP = 9.33 mg/l (normal range < 0,5 mg/l), indicating a possible surgical site infection. On the sixth post-operative day, the patient underwent a washout and surgical debridement of the surgical wound and replacement of the femoral component with another. Tissue samples were sent for culture and histopathological examination. The patient was started on cefepime i.v. awaiting the microbiological results. The results of cultures for aerobic and anaerobic bacteria and acid-resistant bacteria were negative (Fig. 1).

As there was no improvement on the 13th-post-operative day, an infectious-disease specialist consultation was requested, and a triple antibiotic medication was introduced to the patient's medication (tigecycline-ceftolozane-isavuconazole). A dermatological consultation was also requested and considering the clinical features of the ulcer, the absence of infection and the findings of the histopathological examination, the diagnosis of the pyoderma gangrenosum was established. High dose of corticosteroids was introduced to the patient's medication. Also, a vacuum-assisted-closure device was initially used, followed by wound closure in the operating room. Tissue samples from above and below the fascia lata were collected and sent for culturing. The results of cultures for aerobic and anaerobic bacteria and acid-resistant bacteria revealed *S. aureus* and *Candida albicans*, so fungustatin was initiated to her medication.

Gradually, there was an improvement of the surgical wound. Nevertheless, after two months of hospitalization, the patient presented other complications with manifestations from other systems. Initially, she presented two episodes of paroxysmal atrial fibrillation, which were treated with digoxin. On the fourth month of hospitalization, she presented acute calculous cholecystitis and was treated successfully conservatively, with fasting, intravenous fluids and antibiotics, and gradual feeding per os. Her antibiotic medication was altered including daptomycin and cefepime. During her sixth month of hospitalization, she presented episodes of seizures confirmed by electroencephalogram. Brain CT was normal. Levetiracetam and valproate acid were introduced in her medication and cefepime was excluded.

During the last month of her hospitalization, the patient presented gradually deteriorating pancytopenia, gradually deteriorating (WBC = 3.200, PLTs = 7.000, Hgb = 6.8), which was attributed to her medication with levetiracetam, valproate acid and daptomycin. A hematological consultation was requested. Despite discontinuing daptomycin and valproate acid, adjusting the levels of levetiracetam and transfusing with red blood cells and platelets, according to the patient's needs, the pancytopenia did not improve and the patient passed away.

## Discussion

The diagnosis of pyoderma gangrenosum is based on clinical suspicion and is validated histopathologically. Post-surgical PG is a rare pathology that develops usually in the first 15 days post-operatively [6]. It most frequently affects women between the second and fifth decade and after lower limb surgery (77.4 %). In 32.3 % of cases, PG is associated with a systemic inflammatory disease, which makes this population at risk [7]. PG is frequently misdiagnosed as an infection of the surgical site, but surgical debridement can be very harmful because of pathergy. Recommended treatment consists of systemic corticosteroid or cyclosporine therapy, with a clinical improvement observed in 24 h [8]. Other options showed satisfying results, such as tacrolimus, azathioprine, mycophenolate mofetil and polyvalent immunoglobulins, but these were experimental treatments on small number of patients. Local application of topical corticosteroids in patients with non-complicated PG not necessitating systemic therapy and with small cutaneous lesions could be efficient for ulcer healing. Improved patient quality of life is the main benefit of this topical treatment, as there are few side effects.

PG is frequently misdiagnosed, leading to a delay in the appropriate medication with corticosteroids. According to our findings from the literature review and our clinical experience, the use of antibiotics in the patient's medication offers no benefit. Some cases do indeed need antibiotical therapy as there is an infection compromise. The use of systemic corticosteroids is considered to be the first-line treatment for PG, as oral prednisone (0.5–1 mg/kg/day) or intravenous corticosteroid (1000 mg/day) and signs of improvement can be seen within two-three days. According to our clinical experience, despite improvement of the surgical wound, the patient can suffer from many complications following the initial misdiagnose and use of multiple medications. So, it is essential for the surgeon to be alerted and bear in mind the clinical suspicion of the PG, so the appropriate medication is initiated.

## Funding

There are no funding sources.

## Declaration of competing interest

The authors state that there is no conflict of interest.
